# Delayed respiratory syncytial virus outbreak in 2020 in Taiwan was correlated with two novel RSV‐A genotype ON1 variants

**DOI:** 10.1111/irv.12951

**Published:** 2021-12-16

**Authors:** Chun Yi Lee, Tsung Hua Wu, Yu Ping Fang, Jih Chin Chang, Hung Chun Wang, Shou Ju Lin, Chen Hao Mai, Yu Chuan Chang, Teh Ying Chou

**Affiliations:** ^1^ Department of Pediatrics Chang Bing Show Chwan Memorial Hospital Changhua Taiwan; ^2^ Institute of Clinical Medicine National Yang Ming Chiao Tung University Taipei Taiwan; ^3^ Department of Pediatrics Show Chwan Memorial Hospital Changhua Taiwan; ^4^ Department of Pathology and Laboratory Medicine Taipei Veterans General Hospital Taipei Taiwan

**Keywords:** epidemiology, genotype, respiratory syncytial virus, Taiwan

## Abstract

**Background:**

Human respiratory syncytial virus (RSV) is a leading pathogen of acute respiratory tract disease among infants and young children. Compared with previous seasons, RSV outbreaks in Taiwan during the 2020–2021 season were delayed because of COVID‐19 mitigation measures. We conducted this study to determine the association of viral factors with clinical characteristics of preschool children with RSV infection.

**Methods:**

We performed a molecular epidemiology analysis of RSV among inpatient preschool children in Taiwan. In 80 nasopharyngeal samples positive for RSV, we sequenced and analyzed viral genotypes according to patient data. Patients' clinical data were obtained from medical files, and their clinical profiles were compared with those of RSV cases recorded during the 2014–2017 seasons.

**Results:**

Phylogenetic analysis revealed that among the RSV‐positive samples, all RSV strains identified during the 2020–2021 season belonged to the ON1 genotype. Most of the Taiwan ON1 strains were categorized into two well‐supported clusters with distinct G protein amino acid substitution patterns that had never been demonstrated previously. Furthermore, the proportion of cases among children aged >24 months increased (*P* < 0.001). Compared with patients infected during the 2014–2017 seasons, patients infected during the 2020–2021 season were hospitalized for shorter days from hospital admission to dereference (*P* = 0.004) and had a greater need for oxygen supplements (*P* = 0.021) and systemic steroid therapy (*P* = 0.026).

**Conclusion:**

The delayed 2020–2021 RSV outbreak in Taiwan was caused by two novel RSV ON1.1 variants. How the change in RSV epidemiology affects future RSV outbreaks warrants exploration.

## 1 INTRODUCTION

Respiratory syncytial virus (RSV) is a leading cause of severe lower respiratory tract infections in children aged <5 years of age worldwide. Most children have at least one episode of RSV infection by age 2, but repeated infections are common and can lead to severe diseases in the elderly people and high‐risk adults.^1^, ^2^ RSV is the leading viral pathogen of childhood community acquired pneumonia.^3^, ^4^ According to one global surveillance study in 2015, RSV infection was attributed to 33.1 million episodes of lower respiratory tract infection, 3.2 million hospitalizations, and as many as 118,200 deaths.^5^

RSV belongs to the Pneumoviridae family, *Orthopneumovirus* genus, and consists of a single‐stranded, negative sense RNA genome packaged in a lipid envelope. The RSV genome is approximately 15.2 kb in length and encodes 11 proteins. The external glycoproteins F and G are two primary antigenic sites and vital elements for viral attachment.^6^ The G protein of RSV is highly glycosylated and contributes to immune evasion and antigenicity.^7^ The ectodomain of the G protein contains two hypervariable regions (HVR) spanning a length of 13 amino acids in the central conserved cysteine‐rich domain, and its size ranges from 282 to 321 amino acids. The G protein, especially second HVR, is highly diverse and under selection pressure.^8^ These genetic and antigenic variations in the protein are used for the molecular characterization of RSV strains. RSV strains have been classified into two subgroups, namely, A and B (RSV‐A and RSV‐B, respectively), and at least 13 HRSV‐A genotypes and 20 HRSV‐B genotypes have been identified on the basis of G gene sequences.^9^ Although disease severity has been reported to be correlated with specific strains or genotypes,^10^, ^11^, ^12^, ^13^ no consistent association has been established yet.^14^

The extensive use of strict nonpharmaceutical interventions in 2020 to combat the COVID‐19 pandemic changed the RSV circulation pattern and engendered a delay in the annual RSV outbreak in several countries.^15^, ^16^, ^17^ In general, RSV infection occurs biennially with peaks in spring and fall in Taiwan.^18^ The RSV genotypes ON1 and BA9 have co‐circulated in Taiwan with alternating predominance since 2011.^19^ Amid the threat of the COVID‐19 pandemic, an extremely low level of RSV activity was observed in the spring of 2020 in Taiwan, followed by a delayed outbreak at the end of the year. Accordingly, in this study, we explored the clinical features and genotype evolution of this delayed RSV outbreak in Taiwan and evaluated the effect of RSV genetic changes on clinical presentations.

## 2 MATERIALS AND METHODS

### 2.1 Study design and sample collection

We conducted a respiratory pathogen surveillance study on children aged <4 years who were hospitalized for wheezing at Chang Bing Show Chwan Memorial Hospital and Show Chwan Memorial Hospital in Taiwan between October 2019 and February 2021. A nasopharyngeal swab was collected from each child after informed consent was obtained from the child's parents or legal guardian; all samples were placed in a viral transport medium and stored at −80°C before further analysis. Children with a malignancy, immunodeficiency, neurological disability, or cardiopulmonary disease were not enrolled in this study. Demographic information and medical data for each patient were collected manually from electronic records. This study was approved by the Local Ethics Committee of Show Chwan Memorial Hospital (IRB 1081002).

To determine the effect of RSV strains on clinical features, we used 48 RSV‐A cases from our previous study on child wheezing during the 2014–2017 seasons as a comparison.

### 2.2 RNA extraction, cDNA synthesis, RSV detection, and G protein sequencing

Total viral RNA was extracted using a QIAamp viral RNA minikit (Qiagen, Valencia, CA, USA) according to the manufacturer's instructions. Reverse transcription (RT) was performed using random primers and an MMLV RT kit (Protech Technology Enterprise Co, Ltd, ROC). RSV was confirmed through a multiplexed TaqMan RT‐polymerase chain reaction (PCR) analysis of the RSV‐N gene using RSV‐A‐ and RSV‐B‐specific primer/probe mixes on the respiratory nasopharyngeal samples.^20^ Additionally, we screened for the presence of human rhinovirus and *Mycoplasma pneumoniae* on all samples through PCR tests. The cDNA in RSV‐positive samples was amplified using a nested PCR test targeting the G ectodomain region, and the amplicons underwent Sanger sequencing executed using an ABI 3730 automated sequencer (Applied Biosystems). The representative sequences in this study were uploaded to GenBank.

### 2.3 Phylogenetic and amino acid substitution analysis

Nucleotide sequences of the G genes of RSV with determined genotypes were obtained from GenBank for reference. Sequences derived in our study were aligned with reference sequences by using the MUSCLE program implemented in MEGA7 software. Phylogenetic trees were inferred using the neighbor‐joining method implemented in MEGA7, and 1000 replications of bootstrap probabilities were used to evaluate confidence estimates.

We compared the complete sequences of the G genes of all RSV‐positive samples from our two studies with representative G gene sequences obtained from GenBank for the periods 2015 and 2020. Deduced amino acid sequences were translated with standard genetic code using MEGA 7 software. Mutations were determined through comparisons of the strains with the corresponding prototype strains. The mutations of the RSV‐ON1 strains in this study were described with respect to their prototype strains: ON67‐1210A (accession number JN257693).

### 2.4 Statistical analysis

Statistical analysis was performed using SPSS (IBM SPSS Statistics version 22). Descriptive data are presented as proportions. Continuous data are presented as mean ± standard deviation (SD) and analyzed through an independent t test. Group comparisons were performed through either a chi‐square test or Fisher's exact test. Results with a *P* value of <0.05 were considered significant.

## 3 RESULTS

### 3.1 RSV epidemiological prevalence

**FIGURE 1:**
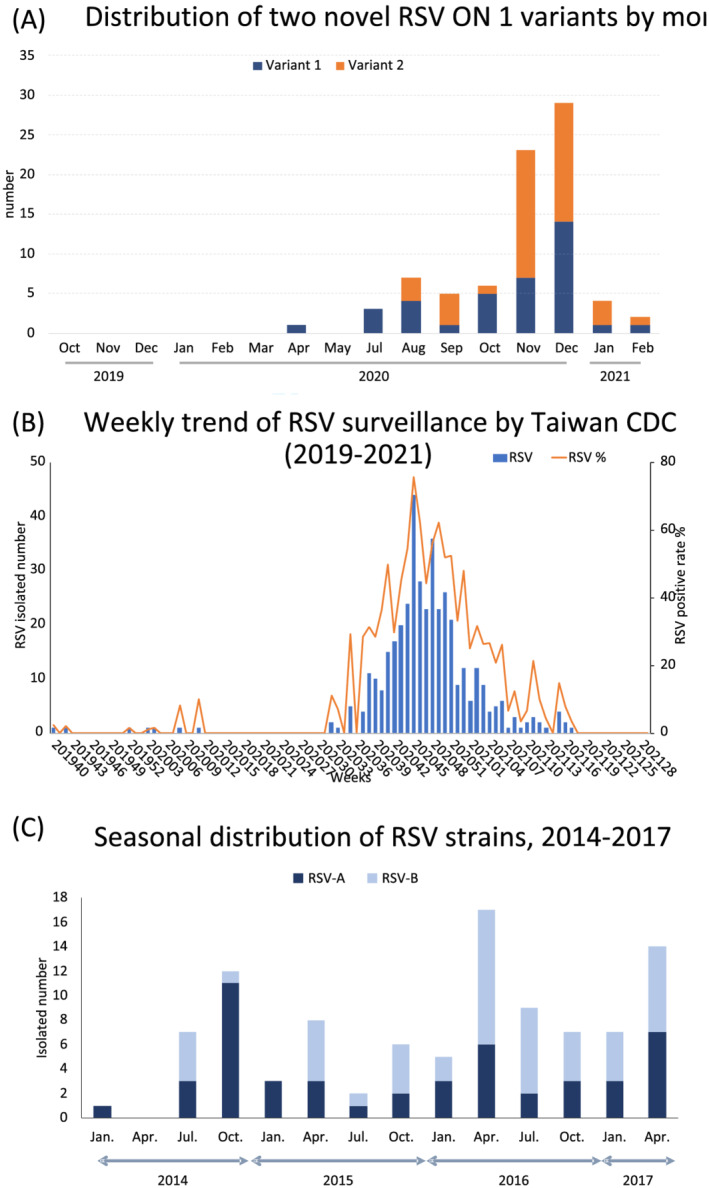
Distribution of respiratory syncytial virus (RSV) detected throughout the study period. (A) Monthly distribution of detected RSV in the present study. (B) Weekly trend of RSV strains isolated in Taiwan from week 40, 2019, to week 28, 2021. This figure was modified according to the database from the Taiwan centers for disease control website (https://nidss.cdc.gov.tw/Home/Index?op=2). (C) Seasonal distribution of identified RSV strains during 2014–2017 seasons

The monthly distribution of identified RSV cases throughout the study period is displayed in Figure 1A. Because of the COVID‐19 pandemic, RSV activity was extremely low in Taiwan until September 2020, when an increase of RSV cases was noted. An upsurge occurred in December 2020 and started to fade in February 2021. This trend in RSV detections is consistent with that recorded on the Taiwan Centers for Disease Control's respiratory viral surveillance database (Figure 1B). Of the 128 enrolled patients in the study, 80 were confirmed to have RSV infection, and real‐time RT‐PCR revealed that all RSV isolates were RSV‐A.

### 3.2 Demographic and clinical profiles of RSV cases of 2020/2021 season

The complete demographic and clinical profiles of the 80 RSV cases are presented in Table 1. The mean age of the patients with RSV infection was 24.5 ± 15.6 months (median age, 26 months; interquartile range [IQR], 10–38 months), and 47 of them were boys. Regarding the distribution of cases according to clinical diagnoses, 30 patients had acute bronchiolitis or bronchitis, 37 had bronchopneumonia, and 23 had viral pneumonia. Compared with that observed during the 2014–2017 seasons, the mean age of patients with RSV infection was higher during the 2020–2021 season (24.5 vs. 13.2 months, *P* < 0.001), and the proportion of patients aged >2 years was significantly higher during the 2020–2021 season (41/80 vs. 7/48, *P* < 0.001). Clinical features, namely, days from hospital admission to dereference, duration of hospitalization, level of oxygen demand, necessity of a bronchodilator, and placement in intensive care, did not differ significantly between the 2014–2017 and 2020–2021 seasons. Patients during the 2020–2021 season, however, had less of a need for supplementary oxygen (*P* < 0.001) and were more often treated with systemic steroids (*P* = 0.025). Moreover, no differences in total white cell count or C‐reactive protein levels were noted, but a higher neutrophil/lymphocyte count ratio was observed in the patients during the 2020–2021 season (*P* < 0.001). We conducted further multivariable analysis by controlling for age and sex. The analysis results revealed that only three parameters differed significantly between the two study seasons: days from admission to dereference (*P* = 0.004), need for supplementary oxygen (*P* = 0.021), and systemic steroid therapy (*P* = 0.026; Table 2).

**TABLE 1:** Demographic characteristics of children with RSV LTRI in 2020–2021 compared with those of children with RSV LRTI in the 2015–2017 seasons

	2020/2021	2014/2017	*P* value
Total case number	80	48	
Male	47 (58.7%)	37 (77.1%)	0.030
Age (months, mean ± SD)	24.7 ± 15.5	13.2 ± 8.9	<0.001
Age ≤24 months	39	41	<0.001
Age >24 months	41	7	
Diagnosis			<0.001
Acute bronchiolitis/bronchitis	30 (37.5%)	48 (100%)	
Bronchopneumonia	23 (28.6%)	0 (0.0%)	
Viral pneumonia	27 (33.8%)	0 (0.0%)	
Clinical manifestations			
Duration of fever (day)	2.7 ± 2.6	3.5 ± 2.8	0.095
Hospitalization duration (day)	5.4 ± 2.1	5.6 ± 2.1	0.642
Oxygen demand	29 (36.3%)	35 (72.9%)	<0.001
Systemic steroid use	24 (30%)	6 (12.5%)	0.025
Bronchodilator use	53 (66.3%)	23 (47.9%)	0.062
Intensive care	12 (15.2%)	5 (10.4%)	0.444
Laboratory findings			
White cell count (× 10^4^/ul)	9763.3 ± 4266.5	10324.2 ± 3379.6	0.389
N/L ratio	1.9 ± 2.0	1.0 ± 0.9	0.007
CRP (mg/dl)	1.1 ± 1.7	1.2 ± 2.4	0.945

Abbreviations: CRP, C‐reactive protein; N/L ratio, neutrophil/lymphocyte count ratio; RSV, respiratory syncytial virus.

**TABLE 2:** Multivariate analysis of clinical features of RSV LTRI during two RSV study seasons

	2020/2021	2014/2017	*P* value
Total case number	80	48	
Clinical manifestations			
Duration of fever (day)	2.5 ± 0.3	4.0 ± 0.4	0.004
Hospitalization duration (day)	5.5 ± 0.2	5.6 ± 0.3	0.803
Oxygen demand	29 (36.3%)	35 (72.9%)	0.021
Systemic steroid use	24 (30%)	6 (12.5%)	0.026
Bronchodilator use	53 (66.3%)	23 (47.9%)	0.523
Intensive care	12 (15.2%)	5 (10.4%)	0.373
Laboratory findings			
White cell count (× 10^4^/ul)	9932.4 ± 461.7	10551.3 ± 619.5	0.430
N/L ratio	1.7 ± 0.2	1.6 ± 0.2	0.823
CRP (mg/dl)	1.0 ± 0.2	1.4 ± 0.3	0.326

Abbreviations: CRP, C‐reactive protein; N/L ratio, neutrophil/lymphocyte count ratio; RSV, respiratory syncytial virus.

Multivariable analysis controlled for age and sex, and data are presented as marginal mean ± standard error. Continuous data were analyzed using a general linear model; categorical data were analyzed using logistic regression.

### 3.3 Phylogenetic analysis of RSV

**FIGURE 2:**
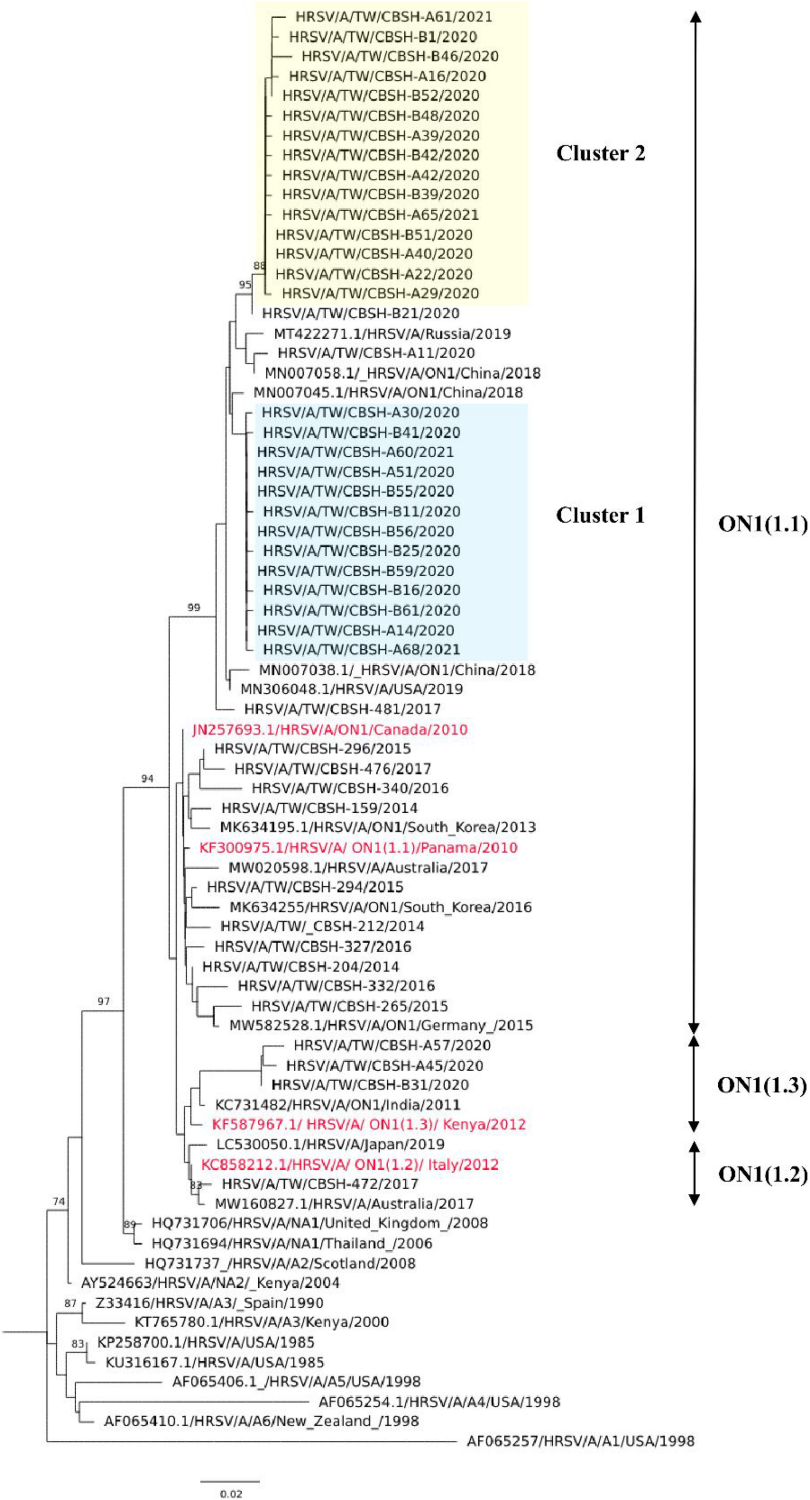
Phylogenetic tree of respiratory syncytial virus (RSV)‐A strains in Taiwan during 2020–2021 season. Phylogenetic tree of unique RSV genotype ON1 G gene ectodomain sequences, which was constructed with the neighbor‐joining method and 1000 replicates for the bootstrap test using MEGA7 software. Only bootstrap values >70 were considered significant and are presented at the branch nodes. Two novel ON1.1 variants circulating during the study season are highlighted

The ectodomain of RSV‐G gene representative sequences was successfully sequenced in 78 of the 80 RSV isolates and deposited to GenBank (accession numbers MZ747620‐MZ747652). Phylogenetic analysis suggested that all RSV‐A strains belonged to the ON1 genotype. The global circulating RSV ON1 genotype can be divided into three lineages, namely, ON1‐1.1, ON1‐1.2, and ON1‐1.3, and the RSV strains obtained during the 2020 and 2014–2017 seasons were located in the ON1‐1.1 lineage. The prevailing RSV ON1 strains observed during the 2020–2021 season diverged from the older circulating RSV ON1 strains observed during the 2014–2017 seasons and were categorized into two clusters (Figure 2). Genetically, the closest global strains to cluster 1 were a 2019 Russian strain (accession number MT422271.1) and a 2018 China strain (accession number MN007058.1); moreover, the closest strains to cluster 2 were a 2018 China strain (accession number MN007045.1) and a 2017 China strain (accession number MW260584.1).

### 3.4 Deduced amino acid sequence analysis

**FIGURE 3:**
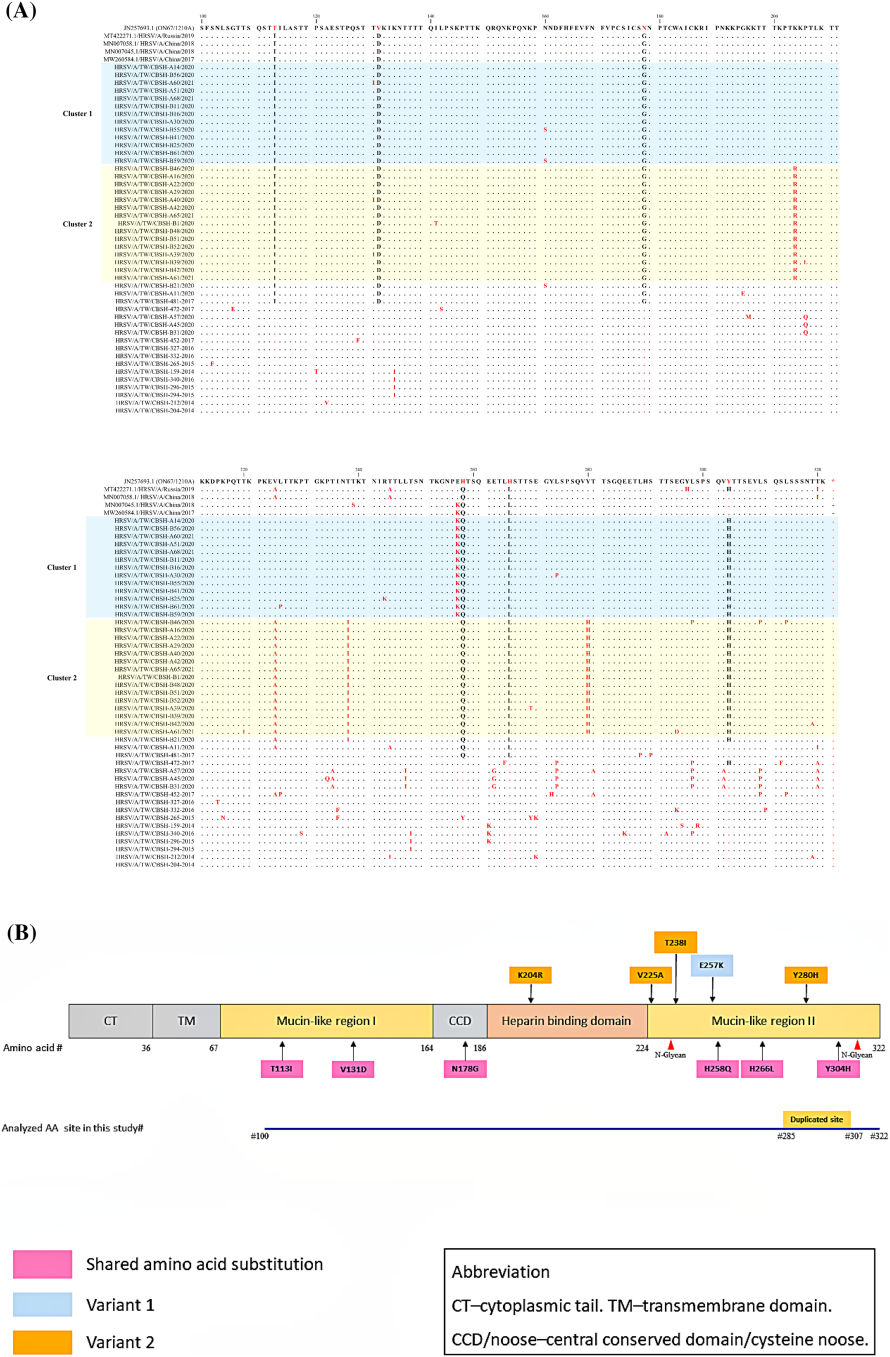
Deduced amino acids of representative respiratory syncytial virus (RSV) ON1 G protein during 2020–2021. (A) Seventy‐eight representative ON1 amino acid sequences corresponding to positions 100 to 322 of the G protein ectodomain were aligned with the prototype ON1 strain ON67‐1210A (JN257693). (B) Summary of the signature G protein amino acid substitutions of two circulating RSV ON1 genotype variants in Taiwan in the 2020–2021 season

The ectodomain of the G protein amino acid sequences obtained from the 78 available RSV strains, spanning 100 to 320 amino acids, was aligned with the ON1 reference strain (accession number JN257693; Figure 3A). Seventy‐five strains could be grouped into two clusters based on the pattern of amino acid substitutions. A total of six shared mutations were noted among these 75 strains: T113I, V131D, N178G, H258Q, H266L, and Y304H. In addition to these amino acid changes, cluster 1 strains specifically harbored an E257K substitution, and cluster 2 strains had a fixed set of genetic alterations of K204R, V225A, T238I, and Y280H. These amino acid substitutions observed in the 2020 strains were distinct from those observed in the older RSV ON1 strains in Taiwan, which comprised E262K, L274P, L298P, and P300S as the prevailing genetic alterations (unpublished data). We designated the RSV strains responsible for clusters 1 and 2 as ON1.1 genotype variants 1 and 2, respectively; Figure 3B presents the changes in their amino acid sequences and their relative positions on the RSV‐G protein sequence.

### 3.5 Comparison of clinical characters between two groups infected ON1 variants

We observed 44 patients with variant 1 infection and 31 patients with variant 2 infection. Table 3 presents a comparison of the clinical characteristics of these two groups. These groups were similar in age, sex, and clinical diagnosis. The groups did not differ significantly in duration of fever and hospitalization, necessity of oxygen or intensive care, use of steroids and antibiotics, or laboratory findings. However, patients with variant 2 infection had a higher rate of bronchodilator use (*P* = 0.02).

**TABLE 3:** Comparison of clinical features of two groups of patients infected by RSV ON1 variants

	Variant 1	Variant 2	*P* value
Total case number	44	31	
Age (months, mean ± SD)	24.6 ± 15.8	23.5 ± 15.5	0.76
Diagnosis			
Acute bronchiolitis/bronchitis	18 (40.1%)	11 (35.4%)	0.74
Bronchopneumonia	10 (22.7%)	11 (35.4%)	
Viral pneumonia	16 (36.4%)	9 (29%)	
Clinical manifestations			
Duration of fever (day)	3.1 ± 2.7	2.3 ± 2.4	0.24
Hospitalization duration (day)	5.3 ± 2.1	5.5 ± 2.1	0.74
Oxygen supplement	14 (31.8%)	12 (38.7%)	0.68
Systemic steroid use	12 (27.2%)	11 (35.4%)	0.31
Bronchodilator use	24 (54.5%)	26 (83.8%)	0.02
Intensive care	3 (6.8%)	7 (22.5%)	0.12
Antibiotics use	22 (50%)	14 (45.1%)	0.11
Laboratory findings			
White cell count (× 10^4^/ul)	9919.5 ± 4502.6	10074.5 ± 4034.3	0.87
N/L ratio	1.8 ± 1.8	1.8 ± 2.4	0.96
CRP (mg/dl)	1.3 ± 2.0	0.7 ± 1.0	0.16

*Note*: Variant 1 featured as E257K substitution. Variant 2 featured as set of K204R/E224A/T238I/Y280H substitutions.

Abbreviations: CRP, C‐reactive protein; N/L ratio, neutrophil/lymphocyte count ratio; RSV, respiratory syncytial virus.

## 4 DISCUSSION

Herein, we describe the epidemiology, clinical features, and genetic alterations of RSV during the 2020–2021 season in Taiwan. COVID‐19 mitigation measures implemented in 2020 engendered changes in the epidemiology of RSV infection. RSV infection in Taiwan usually occurs biennially, with peaks in spring and fall. However, a delayed RSV epidemic occurred in the winter of 2020. Phylogenetic analysis revealed that all RSV strains identified in the 2020 season belonged to RSV‐A genotype ON1. In general, RSV serogroups A and B cocirculate in the community, and the RSV‐A ON1 and RSV‐B BA genotypes have become the two predominant strains globally since their emergence in 2010 and 1999, respectively. According to Chi et al. and our own unpublished data, genotype BA9 began prevailing in Taiwan in 2009; RSV genotype ON1, however, emerged in 2011 and has been the predominant RSV‐A type since 2013. These two genotypes exhibited alternating predominance, and the BA9 genotype dominated during the 2016–2017 season.

Of the RSV proteins, the attachment G protein has the highest genetic diversity and exhibits continual changes.^8^, ^9^ The highest frequency of amino acid substitutions was observed in the second HVR of the G protein. Along with the gain or loss of glycosylation, these amino acid changes contribute to the evolution of G antigenic sites and presumably facilitate the weakening of preexisting immunity. The RSV genotype ON1 continues to evolve locally and globally.^21^ Studies have described several E262K, L274P, L298P, P300S, Y304H, and L310P substitutions in viruses,^22^, ^23^, ^24^including in the RSV strains in Taiwan during the 2014–2017 seasons.

During the 2020–2021 RSV season, we found two RSV genotype ON1 variants cocirculating in Taiwan, which were genetically close to the China strains observed during 2018 and 2019. These variants shared six signature amino acid changes: T113I, V131D (mucin like region 1), N178G (central conserved domain), H258Q, H266L, and Y304H (mucin like region 2). These changes, except forY304H, were identical to those observed in the China strains during 2018 and 2019.^10^ RSV ON1 was found in two Taiwanese variants: one of the Taiwan variants had an additional E257K change, and the other acquired a set of four amino acid substitutions (K204R, V225A, T238I, and Y280H). These substituted amino acids are located within known antigenic sites of the G protein^25^, ^26^ and are distinct from previous RSV strains in Taiwan. Phylogenetic analysis also revealed that the 2020 circulating RSV strains diverged from previous local Taiwan strains.

Compared with RSV cases observed during the 2014–2017 seasons, the atypical age distribution for patients with RSV infection during the 2020 RSV season was notable. This phenomenon was also observed in the 2020 RSV season in France, which showed an increase in the proportion of cases among children aged 3 months to 5 years.^16^ Human humoral and cellular immunity only confers partial and nondurable protection against subsequent RSV infection.^27^, ^28^ RSV antigenic changes, in addition to glycosylation alterations, may have also reduced the effectiveness of preexisting immunity.^11^, ^28^, ^29^

Whether an RSV genotype or variant affects disease severity warrants exploration. Several studies have determined that certain RSV genotypes are linked to increased disease severity and a higher rate of lower respiratory tract infections compared with other RSV genotypes; nevertheless, the mechanism underlying these findings remains inconclusive.^12^, ^22^, ^30^ Patients with RSV in our study had a great need for bronchodilator use and systemic steroid therapy during the 2020–2021 season. However, because of our limited case number and lack of a clinical severity assessment, we could not determine whether the variation of circulating ON1 strains has affected the disease's clinical severity.

Few studies have explored the correlations between RSV variants and clinical severity. Vos et al. determined that an RSV ON1 variant with eight novel substituted amino acids was associated with relatively high disease severity during the 2016–2017 season in the Netherlands.^29^ Another study reported that RSV ON1 variants circulating in the 2017–2018 season in Rome, Italy, caused more severe bronchiolitis.^11^ In addition to novel amino acid changes, changes in the number of O‐ and N‐glycosylation sites of the G protein were observed in these two ON1 variants,^25^, ^29^ which may contribute to changes in immunogenicity and viral virulence. However, another report from China demonstrated that a novel RSV ON1 variant in the 2018–2019 season, which was genetically close to the Taiwan strains, caused milder symptoms.^10^ In the present study, we observed several differences between the two study periods in terms of the necessity of supplementary oxygen and the rate of steroid use in patients with RSV infection. Patient age, clinical entity, and history of recurrent wheezing may be possible confounding factors. However, we assumed that the clinical severity levels were comparable between the periods because the periods did not differ in terms of hospital duration or need for intensive care.

The major limitation of the current study is its relatively small sample size. Nevertheless, the study findings demonstrate that the timing of RSV outbreaks was delayed, which was attributed to COVID‐19 mitigation measures and that the evolution of the ON1 strain continued locally. Understanding the selective advantages of these evolving changes, however, requires further research, including immunologic research

In conclusion, this study demonstrated that two novel RSV ON1.1 variants were correlated with a delay in RSV outbreaks during the 2020–2021 season in Taiwan. The delay was related to measures implemented in response to COVID‐19. How the change in RSV epidemiology affects future RSV outbreaks warrants further research. For a clearer understanding of RSV epidemiology, immunology, and evolution, continual tracking of RSV epidemiology is vital.

## FUNDING INFORMATION

This study was funded by Chang Bing Show Chwan Memorial Hospital (RA20019).

## ETHICS STATEMENT

This study was approved by the Local Ethics Committee of Show Chwan Memorial Hospital (IRB 1081002).

## AUTHOR CONTRIBUTIONS

**Chun Yi Lee:** Conceptualization; data curation; formal analysis; funding acquisition; investigation. **Tsung Hua Wu:** Data curation; investigation. **Yu Ping Fang:** Formal analysis; methodology; project administration. **Hung Chun Wang:** Investigation; resources. **Chen Hao Mai:** Investigation; resources. **Yu Chuan Chang:** Investigation; resources. **Teh Ying Chou:** Supervision.

### PEER REVIEW

The peer review history for this article is available at https://publons.com/publon/10.1111/irv.12951.

## Data Availability

The authors confirm that the data supporting the findings of this study are available within the article.
